# Bis(μ-2-hy­droxy­methyl-2-methyl­propane-1,3-diolato)bis­[di­chlorido­titanium(IV)] diethyl ether disolvate

**DOI:** 10.1107/S1600536813031504

**Published:** 2013-11-23

**Authors:** Alastair J. Nielson, Chaohong Shen, Joyce M. Waters

**Affiliations:** a Chemistry, Institute of Natural and Mathematical Sciences, Massey University at Albany, PO Box 102904 North Shore Mail Centre, Auckland, New Zealand; b Chemistry, Institute of Fundamental Sciences, Massey University at Albany, PO Box 102904 North, Shore Mail Centre, Auckland, New Zealand

## Abstract

The title complex, [Ti_2_Cl_4_{CH_3_C(CH_2_O)_2_(CH_2_OH)}_2_], lies across a centre of symmetry with a diethyl ether solvent mol­ecule hydrogen bonded to the –CH_2_OH groups on either side of it. The Ti^IV^ atom is coordinated in a distorted octa­hedral geometry by a tripodal ligand and two terminal chloride atoms. There are three coordination modes for the tripodal ligand distinguishable on the basis of their very different Ti—O bond lengths. For the terminal alkoxo ligand, the Ti—O distance is 1.760 (1) Å, the asymmetric bridge system has Ti—O bond lengths of 1.911 (1) and 2.048 (1) Å. The Ti—O bond length for the alcohol O atom is the longest at 2.148 (1) Å.

## Related literature

For general background to Ti—O and Ti—Cl bonds, see: Gau *et al.* (1996 ▶); Wu *et al.* (1996 ▶). For closely related structures, see: Talbot-Eeckelaers *et al.* (2006 ▶); Chang *et al.* (1993 ▶); Salta & Zubieta (1997 ▶); Chen *et al.* (1997 ▶). For cluster compounds of this ligand type, see: Boyle *et al.* (1995 ▶); Delmont *et al.* (2000 ▶); Liu *et al.* (1990 ▶).
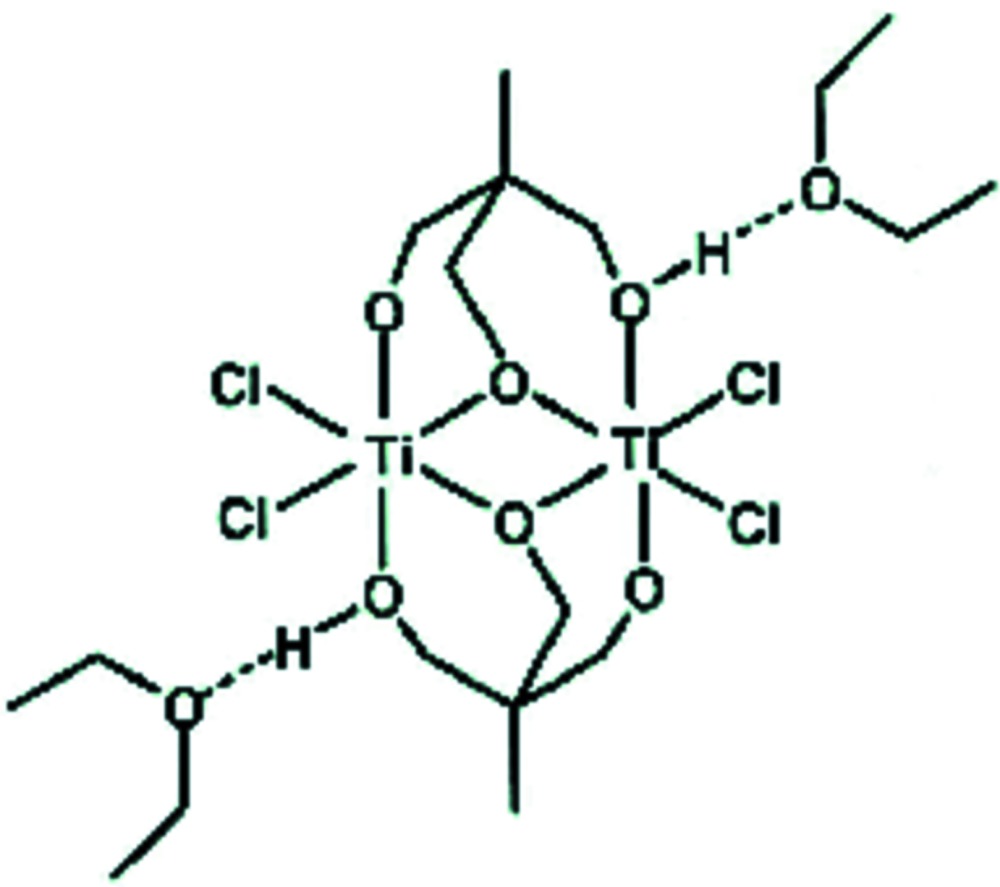


## Experimental

### 

#### Crystal data

[Ti_2_Cl_4_(C_5_H_10_O_3_)_2_]·2C_4_H_10_O
*M* *_r_* = 622.10
Triclinic, 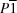

*a* = 7.9617 (3) Å
*b* = 9.6379 (4) Å
*c* = 10.5783 (5) Å
α = 71.351 (1)°
β = 82.023 (1)°
γ = 66.757 (1)°
*V* = 706.60 (5) Å^3^
*Z* = 1
Mo *K*α radiation
μ = 0.98 mm^−1^
*T* = 150 K
0.26 × 0.24 × 0.10 mm

#### Data collection

Siemens SMART CCD diffractometer
Absorption correction: multi-scan (Blessing, 1995 ▶) *T*_min_ = 0.766, *T*_max_ = 0.892
6510 measured reflections
2661 independent reflections
2417 reflections with *I* > 2σ(*I*)
*R*_int_ = 0.015

#### Refinement

*R*[*F* ^2^ > 2σ(*F* ^2^)] = 0.022
*wR*(*F* ^2^) = 0.061
*S* = 1.04
2661 reflections
152 parameters
H atoms treated by a mixture of independent and constrained refinement
Δρ_max_ = 0.31 e Å^−3^
Δρ_min_ = −0.27 e Å^−3^

### 

Data collection: *SMART* (Siemens, 1995 ▶); cell refinement: *SAINT* (Siemens, 1995 ▶); data reduction: *SAINT*; program(s) used to solve structure: *SHELXS97* (Sheldrick, 2008 ▶); program(s) used to refine structure: *SHELXL97* (Sheldrick, 2008 ▶); molecular graphics: *ORTEP-3 for Windows* (Farrugia, 2012 ▶); software used to prepare material for publication: *SHELXL97*.

## Supplementary Material

Crystal structure: contains datablock(s) I, global. DOI: 10.1107/S1600536813031504/hg5360sup1.cif

Structure factors: contains datablock(s) I. DOI: 10.1107/S1600536813031504/hg5360Isup2.hkl

Additional supplementary materials:  crystallographic information; 3D view; checkCIF report

## Figures and Tables

**Table 1:** Hydrogen-bond geometry (Å, °)

*D*—H⋯*A*	*D*—H	H⋯*A*	*D*⋯*A*	*D*—H⋯*A*
O3—H3⋯O11	0.74 (2)	1.89 (2)	2.6233 (14)	168 (2)

**Fig. 1.**
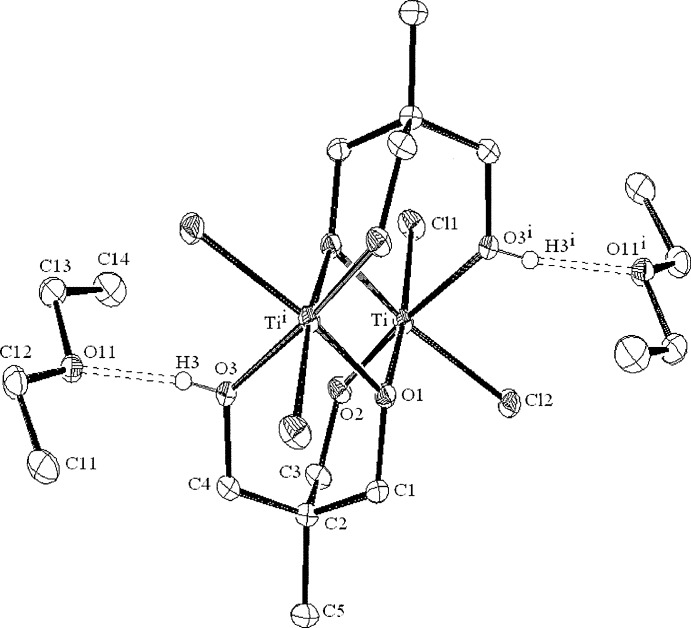
ORTEP diagram of molecule, at the 50% probability level, showing the numbering system. Symmetry code: (i) -x + 1, -y + 1, -z + 1.

## supplementary crystallographic information

### 1. Comment

Transition metal complexes that arise from the *tris*-hydroxymethyl ethane ligand CH_3_C(CH_2_OH)_3_ are not widely reported in the literature. Structures identified by X-ray crystallography include the triply deprotonated form in cluster compounds of molybdenum and tungsten (Liu *et al.* 1990; Delmont *et al.* 2000) and dimeric and cluster compounds of chromium (Talbot-Eeckelaers *et al.* 2006), the doubly deprotonated form in dimeric vanadium-oxo complexes (Chang *et al.* 1993; Salta & Zubieta 1997) and the fully protonated form in monomeric yttrium complexes (Chen *et al.* 1997). For titanium only µ3–O and µ2–O coordination modes have been identified (Boyle *et al.* 1995).

When TiCl_4_ in diethyl ether was reacted with a mixture of CH_3_C(CH_2_OSiMe_3_)_3_ and CH_3_C(CH_2_OSiMe_3_)_2_(CH_2_OH) we obtained a small amount of colourless crystals analysing as TiCl_2_[(OCH_2_)_2_(HOCH_2_)CCH_3_]. 0.5 diethyl ether. One crystal was characterized by X-ray crystallography. The structure consists of a centrosymmetric dimer made up of two [(OCH_2_)_2_(HOCH_2_)CCH_3_] ligands forming a tripodal bridging system across two titanium atoms each of which also contain two terminal chloro ligands. One tripodal ligand is positioned above the two Ti atoms and out towards the rear of the molecule and the other is below and out the front with the two related by an inversion centre. The distorted octahedral geometry about Ti is made from one arm of a terminal alkoxo ligand (O2), another from an alcohol ligand (O3) and a third arm from the bridging alkoxo atoms (O1 and O1^i^). These latter each lie in *trans*-positions to the two *cis*-related chloro ligands.

For the terminal alkoxo ligand the Ti–O2 distance is 1.760 (1) Å which is indicative of strong π-bonding (see later). The asymmetric bridge system has Ti–O bond lengths of 1.991 (1) and 2.048 (7) Å and the Ti–Cl bonds *trans* to these bridging O atoms also show differences in length, 2.3184 (4) Å for Ti–Cl1 which is *trans* to the longer Ti–O1 and 2.3292 (4) Å for Ti–Cl2 which lies *trans* to the shorter Ti–O1^i^ bond. The Ti–O bond length for the alcohol oxygen (Ti–O3) is the longest at 2.148 (1) Å. The strongly π-bonded terminal alkoxo ligand O atoms (O2, O2^i^) lie *trans* to the weak dative bonds made by the alcohol ligand O atoms (O3^i^, O3).

The overall coordination geometry and deprotonation features of the two ligands are identical with that found in the vanadium (v) complex {V_2_O_2_Cl_2_([(OCH_2_)_2_(HOCH_2_)CCH_3_]_2_} (Chang *et al.* 1993; Salta & Zubieta 1997). In {M_2_O_4_[(OCH_2_)_3_CCH_3_]_2_}^2-^ (M = Mo, W) all three arms of the tripod are deprotonated (Liu *et al.* 1990; Delmont *et al.* 2000). Single deprotonation occurs in {Cr_2_Cl_4_([(OCH_2_)HOCH_2_)_2_(CEt]_2_} (Talbot-Eeckelaers *et al.* 2006).

The Ti–O bond lengths in the present complex reflect the various coordination modes in the molecule. The Ti–O1 bond lengths [1.991 (1) and 2.048 (1) Å] show the asymmetric nature of the bridging system across the two Ti atoms. The short Ti–O2 bond length [1.760 (1) Å] is associated with an alkoxo ligand. In comparison, terminal alkoxo ligand Ti–O bond lengths range from 1.702 (4) to 1.742 (6) Å in a variety of *iso*-propoxo Ti complexes (Gau *et al.* 1996). The dative nature of the alcohol ligand is shown by the longer Ti–O3 bond length [2.148 (1) Å] in the present complex being slightly longer than observed for isopropyl alcohol ligated to titanium [bond lengths 2.087 (4) and 2.093 (4) Å] (Wu *et al.* 1996). The Ti–Cl1 and Ti–Cl2 bond lengths [2.3184 (4) and 2.3292 (4) Å] do not differ significantly from each other and are typical of Ti–Cl bonds observed elsewhere. (Gau *et al.* 1996; Wu *et al.* 1996).

The strong π-donor nature of the alkoxo ligand oxygen is shown by the way other coordinated atoms push away from it [O2–Ti–O1, 86.71 (4); O2–Ti–O1^i^, 101.78 (5); O–2–Ti–Cl1, 97.18 (4); O2–Ti–Cl2, 93.95 (4)°]. For the dative Ti–O bond all the associated O3^i^–Ti–Y angles are 90° or below [range 80.17 (4) to 90.02 (3)°]. The bond angle associated with the bridging Ti–O1–Ti^i^ system [103.17 (4)°] does not differ significantly from comparable angles observed in other complexes. In this regard, it is noted that all known complexes have an alkoxo ligand making up the bridging system. The largest terminal Ti–O–C bond angle is associated with the alkoxo ligand [alkoxo Ti–O2–C3 angle 138.33 (9)° *cf.* alcohol Ti^i^–O3–C4 angle 126.66 (9)°]. To accommodate the coordination mode of the various oxygen ligands across the two Ti atoms, the O–C–C bond angles of the tripod [range, 112.4 (1) to 113.3 (1)°] are slightly greater than the ideal tetrahedral angle.

### 2. Experimental

Using normal bench-top techniques for air-sensitive compounds, TiCl_4_ (1.25 g, 6.59 mmol) was cooled to dry-ice temperature and diethyl ether (50 ml) chilled to -20 °C was added. The mixture was warmed to room temperature, heated until all the yellow solid had dissolved and 1,1,1-tris(hydroxymethyl)ethane (2.2 g, 6.6 mmol) in diethyl ether (50 ml) was added to the rapidly stirred solution whereupon a dense colourless precipitate was formed. The mixture was refluxed for 3 h, cooled to room temperature and the remaining solid allowed to settle and the solution was filtered. The volume was reduced to *ca* 30 ml and the solution stood at -20 ° C whereupon a mass of crystalline colourless material was deposited. Found: C, 31.06; H, 6.15%. C_14_H_30_Cl_4_O_6_Ti_2_ (*i.e.* C_10_H_20_Cl_4_O_6_Ti_2_.diethyl ether) requires C, 31.61; H, 5.69%. A crystal was chosen from the mass and the X-ray crystal structure obtained. This molecule corresponded to C_10_H_20_Cl_4_O_6_Ti_2_.*bis*-diethyl ether.

### 3. Refinement

All H atoms (except H3) were included in calculated positions and refined using a riding model with *U*_iso_ = 1.2*U_eq_*(C) for H on secondary C atoms and 1.5*U_eq_*(C) for those on tertiary C atoms. C—H distances of 0.99Å and 0.96Å were assumed for tertiary C and secondary C atoms respectively. H3 was located on a difference map and its *x*, *y*, *z* co-ordinates and isotropic thermal parameter refined.

### Crystal data

**Table:**

[Ti_2_Cl_4_(C_5_H_10_O_3_)_2_]·2C_4_H_10_O	*Z* = 1
*M**_r_* = 622.10	*F*(000) = 324
Triclinic, *P*1	*D*_x_ = 1.462 Mg m^−^^3^
Hall symbol: -P 1	Mo *K*α radiation, λ = 0.71073 Å
*a* = 7.9617 (3) Å	Cell parameters from 5595 reflections
*b* = 9.6379 (4) Å	θ = 2–25°
*c* = 10.5783 (5) Å	µ = 0.98 mm^−^^1^
α = 71.351 (1)°	*T* = 150 K
β = 82.023 (1)°	Plate, colourless
γ = 66.757 (1)°	0.26 × 0.24 × 0.10 mm
*V* = 706.60 (5) Å^3^	

### Data collection

**Table:**

Siemens SMART CCD diffractometer	2661 independent reflections
Radiation source: fine-focus sealed tube	2417 reflections with *I* > 2σ(*I*)
Graphite monochromator	*R*_int_ = 0.015
Area detector ω scans	θ_max_ = 25.7°, θ_min_ = 2.0°
Absorption correction: multi-scan (Blessing, 1995)	*h* = −9→9
*T*_min_ = 0.766, *T*_max_ = 0.892	*k* = −10→11
6510 measured reflections	*l* = 0→12

### Refinement

**Table:**

Refinement on *F*^2^	Primary atom site location: structure-invariant direct methods
Least-squares matrix: full	Secondary atom site location: difference Fourier map
*R*[*F*^2^ > 2σ(*F*^2^)] = 0.022	Hydrogen site location: inferred from neighbouring sites
*wR*(*F*^2^) = 0.061	H atoms treated by a mixture of independent and constrained refinement
*S* = 1.04	*w* = 1/[σ^2^(*F*_o_^2^) + (0.0325*P*)^2^ + 0.2355*P*] where *P* = (*F*_o_^2^ + 2*F*_c_^2^)/3
2661 reflections	(Δ/σ)_max_ < 0.001
152 parameters	Δρ_max_ = 0.31 e Å^−^^3^
0 restraints	Δρ_min_ = −0.27 e Å^−^^3^

### Special details

**Table:**

Geometry. All e.s.d.'s (except the e.s.d. in the dihedral angle between two l.s. planes) are estimated using the full covariance matrix. The cell e.s.d.'s are taken into account individually in the estimation of e.s.d.'s in distances, angles and torsion angles; correlations between e.s.d.'s in cell parameters are only used when they are defined by crystal symmetry. An approximate (isotropic) treatment of cell e.s.d.'s is used for estimating e.s.d.'s involving l.s. planes.
Refinement. Refinement of *F*^2^ against ALL reflections. The weighted *R*-factor *wR* and goodness of fit *S* are based on *F*^2^, conventional *R*-factors *R* are based on *F*, with *F* set to zero for negative *F*^2^. The threshold expression of *F*^2^ > σ(*F*^2^) is used only for calculating *R*-factors(gt) *etc*. and is not relevant to the choice of reflections for refinement. *R*-factors based on *F*^2^ are statistically about twice as large as those based on *F*, and *R*- factors based on ALL data will be even larger.

### Fractional atomic coordinates and isotropic or equivalent isotropic displacement parameters (Å^2^)

**Table:**

	*x*	*y*	*z*	*U*_iso_*/*U*_eq_	
Ti	0.58094 (3)	0.61520 (3)	0.39188 (2)	0.01725 (9)	
Cl1	0.74357 (5)	0.59557 (5)	0.19464 (4)	0.02805 (11)	
Cl2	0.47954 (5)	0.88725 (4)	0.35265 (4)	0.02495 (10)	
O1	0.43149 (13)	0.59722 (11)	0.56614 (9)	0.0177 (2)	
O2	0.76654 (13)	0.57443 (12)	0.48899 (10)	0.0217 (2)	
O3	0.66826 (14)	0.32177 (13)	0.70683 (10)	0.0197 (2)	
C1	0.4634 (2)	0.65978 (17)	0.66438 (14)	0.0209 (3)	
H1A	0.3764	0.6490	0.7400	0.025*	
H1B	0.4384	0.7737	0.6237	0.025*	
C2	0.6586 (2)	0.57709 (17)	0.71785 (15)	0.0211 (3)	
C3	0.7972 (2)	0.60351 (19)	0.60594 (15)	0.0248 (3)	
H3A	0.7897	0.7137	0.5832	0.030*	
H3B	0.9221	0.5328	0.6382	0.030*	
H3	0.721 (3)	0.236 (3)	0.737 (2)	0.038 (6)*	
C4	0.6997 (2)	0.40296 (17)	0.78961 (15)	0.0227 (3)	
H4A	0.8288	0.3514	0.8178	0.027*	
H4B	0.6216	0.3934	0.8709	0.027*	
C5	0.6733 (2)	0.65305 (19)	0.82181 (16)	0.0296 (4)	
H5A	0.5924	0.6323	0.8981	0.044*	
H5B	0.6371	0.7672	0.7813	0.044*	
H5C	0.7997	0.6079	0.8522	0.044*	
O11	0.89033 (14)	0.03405 (12)	0.82316 (10)	0.0220 (2)	
C11	0.7018 (2)	0.0355 (2)	1.01998 (16)	0.0309 (4)	
H11A	0.7672	0.0891	1.0464	0.046*	
H11B	0.6635	−0.0316	1.0999	0.046*	
H11C	0.5939	0.1143	0.9687	0.046*	
C12	0.8258 (2)	−0.06469 (18)	0.93561 (16)	0.0276 (3)	
H12A	0.9306	−0.1496	0.9889	0.033*	
H12B	0.7584	−0.1142	0.9041	0.033*	
C13	1.0030 (2)	−0.0498 (2)	0.73231 (17)	0.0305 (4)	
H13A	0.9318	−0.0910	0.6945	0.037*	
H13B	1.1090	−0.1401	0.7797	0.037*	
C14	1.0679 (2)	0.0633 (2)	0.62220 (18)	0.0375 (4)	
H14A	0.9624	0.1497	0.5732	0.056*	
H14B	1.1492	0.0074	0.5610	0.056*	
H14C	1.1343	0.1064	0.6609	0.056*	

### Atomic displacement parameters (Å^2^)

**Table:**

	*U*^11^	*U*^22^	*U*^33^	*U*^12^	*U*^13^	*U*^23^
Ti	0.01564 (14)	0.01876 (14)	0.01493 (14)	−0.00583 (10)	0.00015 (10)	−0.00266 (10)
Cl1	0.0258 (2)	0.0379 (2)	0.01864 (19)	−0.01292 (16)	0.00543 (15)	−0.00699 (16)
Cl2	0.0292 (2)	0.01963 (18)	0.0251 (2)	−0.01055 (15)	−0.00415 (15)	−0.00208 (14)
O1	0.0167 (5)	0.0178 (5)	0.0160 (5)	−0.0040 (4)	0.0002 (4)	−0.0047 (4)
O2	0.0178 (5)	0.0266 (5)	0.0192 (5)	−0.0073 (4)	−0.0004 (4)	−0.0058 (4)
O3	0.0222 (5)	0.0149 (5)	0.0202 (5)	−0.0041 (4)	−0.0047 (4)	−0.0046 (4)
C1	0.0239 (7)	0.0196 (7)	0.0181 (7)	−0.0051 (6)	0.0006 (6)	−0.0084 (6)
C2	0.0241 (7)	0.0200 (7)	0.0196 (7)	−0.0070 (6)	−0.0026 (6)	−0.0070 (6)
C3	0.0248 (8)	0.0308 (8)	0.0221 (8)	−0.0129 (6)	−0.0025 (6)	−0.0078 (6)
C4	0.0273 (8)	0.0217 (7)	0.0188 (7)	−0.0074 (6)	−0.0051 (6)	−0.0057 (6)
C5	0.0405 (9)	0.0274 (8)	0.0245 (8)	−0.0122 (7)	−0.0050 (7)	−0.0108 (7)
O11	0.0222 (5)	0.0197 (5)	0.0201 (5)	−0.0051 (4)	0.0000 (4)	−0.0042 (4)
C11	0.0341 (9)	0.0376 (9)	0.0224 (8)	−0.0189 (8)	0.0021 (7)	−0.0046 (7)
C12	0.0321 (9)	0.0237 (8)	0.0244 (8)	−0.0124 (7)	−0.0032 (7)	0.0002 (6)
C13	0.0270 (8)	0.0294 (8)	0.0296 (9)	−0.0014 (7)	−0.0008 (7)	−0.0130 (7)
C14	0.0289 (9)	0.0492 (11)	0.0312 (9)	−0.0110 (8)	0.0069 (7)	−0.0151 (8)

### Geometric parameters (Å, º)

**Table:**

Ti—O2	1.7601 (10)	C3—H3B	0.9900
Ti—O1^i^	1.9911 (10)	C4—H4A	0.9900
Ti—O1	2.0478 (10)	C4—H4B	0.9900
Ti—O3^i^	2.1481 (11)	C5—H5A	0.9800
Ti—Cl1	2.3184 (4)	C5—H5B	0.9800
Ti—Cl2	2.3292 (4)	C5—H5C	0.9800
Ti—Ti^i^	3.1649 (5)	O11—C13	1.4374 (19)
O1—C1	1.4501 (17)	O11—C12	1.4413 (18)
O1—Ti^i^	1.9911 (10)	C11—C12	1.502 (2)
O2—C3	1.4243 (18)	C11—H11A	0.9800
O3—C4	1.4473 (18)	C11—H11B	0.9800
O3—Ti^i^	2.1481 (11)	C11—H11C	0.9800
O3—H3	0.74 (2)	C12—H12A	0.9900
C1—C2	1.533 (2)	C12—H12B	0.9900
C1—H1A	0.9900	C13—C14	1.509 (3)
C1—H1B	0.9900	C13—H13A	0.9900
C2—C4	1.523 (2)	C13—H13B	0.9900
C2—C3	1.533 (2)	C14—H14A	0.9800
C2—C5	1.542 (2)	C14—H14B	0.9800
C3—H3A	0.9900	C14—H14C	0.9800
			
O2—Ti—O1^i^	101.78 (5)	C2—C3—H3A	109.1
O2—Ti—O1	86.71 (4)	O2—C3—H3B	109.1
O1^i^—Ti—O1	76.83 (4)	C2—C3—H3B	109.1
O2—Ti—O3^i^	172.39 (5)	H3A—C3—H3B	107.9
O1^i^—Ti—O3^i^	80.17 (4)	O3—C4—C2	112.55 (12)
O1—Ti—O3^i^	86.59 (4)	O3—C4—H4A	109.1
O2—Ti—Cl1	97.18 (4)	C2—C4—H4A	109.1
O1^i^—Ti—Cl1	93.21 (3)	O3—C4—H4B	109.1
O1—Ti—Cl1	169.89 (3)	C2—C4—H4B	109.1
O3^i^—Ti—Cl1	90.02 (3)	H4A—C4—H4B	107.8
O2—Ti—Cl2	93.95 (4)	C2—C5—H5A	109.5
O1^i^—Ti—Cl2	158.75 (3)	C2—C5—H5B	109.5
O1—Ti—Cl2	90.05 (3)	H5A—C5—H5B	109.5
O3^i^—Ti—Cl2	82.46 (3)	C2—C5—H5C	109.5
Cl1—Ti—Cl2	98.956 (16)	H5A—C5—H5C	109.5
O2—Ti—Ti^i^	95.24 (4)	H5B—C5—H5C	109.5
O1^i^—Ti—Ti^i^	39.05 (3)	C13—O11—C12	112.91 (12)
O1—Ti—Ti^i^	37.78 (3)	C12—C11—H11A	109.5
O3^i^—Ti—Ti^i^	81.61 (3)	C12—C11—H11B	109.5
Cl1—Ti—Ti^i^	132.238 (18)	H11A—C11—H11B	109.5
Cl2—Ti—Ti^i^	125.940 (16)	C12—C11—H11C	109.5
C1—O1—Ti^i^	124.13 (8)	H11A—C11—H11C	109.5
C1—O1—Ti	118.13 (8)	H11B—C11—H11C	109.5
Ti^i^—O1—Ti	103.17 (4)	O11—C12—C11	108.69 (13)
C3—O2—Ti	138.33 (9)	O11—C12—H12A	110.0
C4—O3—Ti^i^	126.66 (9)	C11—C12—H12A	110.0
C4—O3—H3	106.5 (16)	O11—C12—H12B	110.0
Ti^i^—O3—H3	117.1 (16)	C11—C12—H12B	110.0
O1—C1—C2	113.32 (11)	H12A—C12—H12B	108.3
O1—C1—H1A	108.9	O11—C13—C14	108.13 (14)
C2—C1—H1A	108.9	O11—C13—H13A	110.1
O1—C1—H1B	108.9	C14—C13—H13A	110.1
C2—C1—H1B	108.9	O11—C13—H13B	110.1
H1A—C1—H1B	107.7	C14—C13—H13B	110.1
C4—C2—C1	110.95 (12)	H13A—C13—H13B	108.4
C4—C2—C3	112.76 (13)	C13—C14—H14A	109.5
C1—C2—C3	110.72 (12)	C13—C14—H14B	109.5
C4—C2—C5	107.06 (12)	H14A—C14—H14B	109.5
C1—C2—C5	108.02 (12)	C13—C14—H14C	109.5
C3—C2—C5	107.07 (13)	H14A—C14—H14C	109.5
O2—C3—C2	112.41 (12)	H14B—C14—H14C	109.5
O2—C3—H3A	109.1		

Symmetry code: (i) −*x*+1, −*y*+1, −*z*+1.

### Hydrogen-bond geometry (Å, º)

**Table:**

*D*—H···*A*	*D*—H	H···*A*	*D*···*A*	*D*—H···*A*
O3—H3···O11	0.74 (2)	1.89 (2)	2.6233 (14)	168 (2)
